# Condor-COPASI: high-throughput computing for biochemical networks

**DOI:** 10.1186/1752-0509-6-91

**Published:** 2012-07-26

**Authors:** Edward Kent, Stefan Hoops, Pedro Mendes

**Affiliations:** 1 Doctoral Training Centre in Integrative Systems Biology, Manchester Institute of Biotechnology, The University of Manchester, 131 Princess Street, Manchester M1 7DN, UK; 2 Virginia Bioinformatics Institute, Virginia Tech, Washington St 0477, Blacksburg, VA 24061, USA; 3 School of Computer Science, Manchester Institute of Biotechnology, The University of Manchester, 131 Princess Street, Manchester M1 7DN, UK

**Keywords:** Systems biology, Computational modelling, High-throughput computing, Distributed computing, Simulation

## Abstract

**Background:**

Mathematical modelling has become a standard technique to improve our understanding of complex biological systems. As models become larger and more complex, simulations and analyses require increasing amounts of computational power. Clusters of computers in a high-throughput computing environment can help to provide the resources required for computationally expensive model analysis. However, exploiting such a system can be difficult for users without the necessary expertise.

**Results:**

We present Condor-COPASI, a server-based software tool that integrates COPASI, a biological pathway simulation tool, with Condor, a high-throughput computing environment. Condor-COPASI provides a web-based interface, which makes it extremely easy for a user to run a number of model simulation and analysis tasks in parallel. Tasks are transparently split into smaller parts, and submitted for execution on a Condor pool. Result output is presented to the user in a number of formats, including tables and interactive graphical displays.

**Conclusions:**

Condor-COPASI can effectively use a Condor high-throughput computing environment to provide significant gains in performance for a number of model simulation and analysis tasks. Condor-COPASI is free, open source software, released under the Artistic License 2.0, and is suitable for use by any institution with access to a Condor pool. Source code is freely available for download at http://code.google.com/p/condor-copasi/, along with full instructions on deployment and usage.

## Background

Mathematical modelling is becoming increasingly recognized as an important tool in the study of biological systems [1]. Databases such as BioModels [2] provide access to peer-reviewed published models, which can be freely downloaded in the standards-compliant SBML format [3]. Model construction, however, is only the first in a number of steps that can be used to gain insight into the function of a complex biological system.

Many models consist of a set of ordinary differential equations (ODEs) describing how the concentrations of various chemical species change with time. The dynamic behaviour of the model can be examined through integration of the ODEs, a relatively computationally simple task. In many cases, it can be helpful to simulate the model behaviour using a stochastic or hybrid algorithm [4], which can yield more information than a deterministic solution can provide. Solving a model using a stochastic method requires more computational power than solving it deterministically [4], and must be repeated many times to yield information on the distribution of potential trajectories.

In addition to examining the dynamic behaviour of a model, we may also wish to analyse it using techniques such as sensitivity analysis – examining the influence of specific model parameters (such as kinetic rate constants) on a systems-level property (such as the flux through the main branch of a pathway). Other tasks commonly used to examine models include optimizations (finding the best set of parameters to maximize or minimize the value of a particular objective function), or parameter fitting (finding the best set of parameters so that the model behaviour most represents that seen experimentally).

### COPASI

A number of software tools are available for biological systems modelling. Some of these, such as MATLAB, are very flexible, but require users to have a knowledge of programming. Other tools, such as COPASI [5], are designed with a user-friendly interface, and do not require any programming expertise, while still providing sophisticated algorithms and analyses.

COPASI allows users to construct and analyse models using a graphical user interface (GUI). Simulations using a deterministic, stochastic or hybrid method can be performed at the click of a button. In addition to time-course simulations, COPASI can perform a range of model analysis tasks, including steady-state analysis, sensitivity analysis, parameter estimation, and optimizations.

As models become more detailed and complex, simulation and analysis requires increasing amounts of computational power, potentially requiring more power than can be offered by even the most high-powered desktop machine.

### Client-server simulation tools

The limitations of using personal computers to perform computationally intensive simulations have led to the development of a number of server-based systems biology tools. These allow a user to set up mathematical models and to display any results on their local machine, but to perform computationally-intensive simulations on one or more remote machines.

One such example is JWS Online [6], a modelling tool accessed through a web-based Java applet, which performs all simulations on a remote server. The computational capacity is limited by the performance of the server, and so it is not ideally suited for computationally intensive tasks. VCell [7] is another tool for model simulation and analysis. Models are prepared using a local Java interface, and simulations are performed on a distributed computing cluster operated by the VCell team, up to a maximum of 100 simulation repeats per submission. Parameter estimations can also be performed, but these are run locally.

Another way of running simulations remotely is to incorporate them into an automated distributed workflow, using a management tool such as Taverna [8]. COPASI Web Services [9] enables COPASI simulation and analysis tasks to be incorporated into a distributed workflow by setting up COPASI simulation servers to offer computing cycles. Using automated workflows allows for very flexible usage patterns, though creating workflows can be a complex process. In addition, users must have the computational resources available for running simulations.

### Condor

Condor is a system for high-throughput computing, allowing computing jobs to be run on a pool of machines [10,11]. The pool can contain dedicated computers, though one of the main strengths of Condor is its ability to utilize non-dedicated machines during periods when they would otherwise be idle. For example, in academic institutions, large numbers of computers are in use during working hours on weekdays, but will sit unused overnight and at weekends. Condor is often configured to detect when a machine is not being used by its owner, and can then assign queued computing jobs to be run on it. Condor is compatible with most major operating systems, including Windows, Linux and OSX.

In order to submit a computing job to run on Condor, a job description file must first be prepared. This file contains information about the executable file to be run, any files that must be transferred to remote machines, and the software and hardware requirements of the job, such as the operating system and minimum amount of memory required. Once the job description has been prepared, it is submitted, and added to a queue for resources. A machine known as the Master decides when, and on which machine each job is to be run.

Condor pools can contain thousands of machines. The computing power of such pools can be most readily exploited when a computing task can be split into multiple, independent, jobs which can be run in parallel. However, the requirement to split large tasks into multiple smaller ones, along with a complex Condor job submission process involving command line tools, makes this difficult for many users.

### Condor-COPASI

COPASI can be used, without any modification, with high-throughput computing environments such as Condor. However, in order to exploit the parallel nature of a Condor pool, any simulations or analyses must be manually split into multiple small, independent jobs a task that can be difficult. In addition, Condor requires the use of command-line tools to submit jobs and to monitor their status. This can be difficult, and may deter many users from making use of these facilities.

Therefore, we developed Condor-COPASI, a tool which integrates COPASI with Condor, allowing users to perform a selection of model analysis and simulation tasks in parallel using a Condor pool. Condor-COPASI is designed to be very simple to use, with all user interaction taking place through a GUI. The process of splitting tasks into an optimal number of parallel jobs, and submitting them to the Condor pool is handled automatically, without requiring user interaction. Once jobs have been submitted to the Condor pool, Condor-COPASI monitors their status, automatically emailing a notification to the user upon completion. Results can be displayed within the interface in a variety of formats, including tables and interactive graphical displays, or can be exported for further processing.

## Implementation

Condor-COPASI is a server-based application, accessed by users through a web interface which is compatible with all modern web browsers. Access to the web interface is controlled on a per-user basis; the administrator must create an account for each user of the system. A database is used to store user account information, along with information about the computing jobs each user has submitted, such the submission and end times, the number of parallel jobs used and CPU hours used. Historical usage data is stored, and statistics can be displayed though the web interface to monitor how the system is being used. Other files, such as COPASI models, and outputted results are stored as flat files in a directory of the system.

We designed Condor-COPASI to run as a server-based application, since the alternative – a local application to be run on each users machine – would require Condor to be installed and configured on each individual local machine. Installing Condor and configuring it to be able to submit jobs is a complicated process, and in many cases impractical. In addition, Condor requires that the machine which is used to submit jobs must remain powered on while remote jobs are running; an onerous requirement for many users, particularly when jobs have a long run time and are submitted from a portable machine. Running as a server-based application removes this requirement for the individual users, since only the server must remain powered on while jobs are running.

Condor-COPASI is written in the Python programming language [12], using the Django web development framework [13]. It must be installed on a server which has access to a Condor pool and permission to submit jobs. Additionally a web server must also be installed (though this is somewhat standard). Finally, a Django-compatible database must be available – choices currently include MySQL, PostgreSQL, Oracle and SQLite.

**Figure 1:**
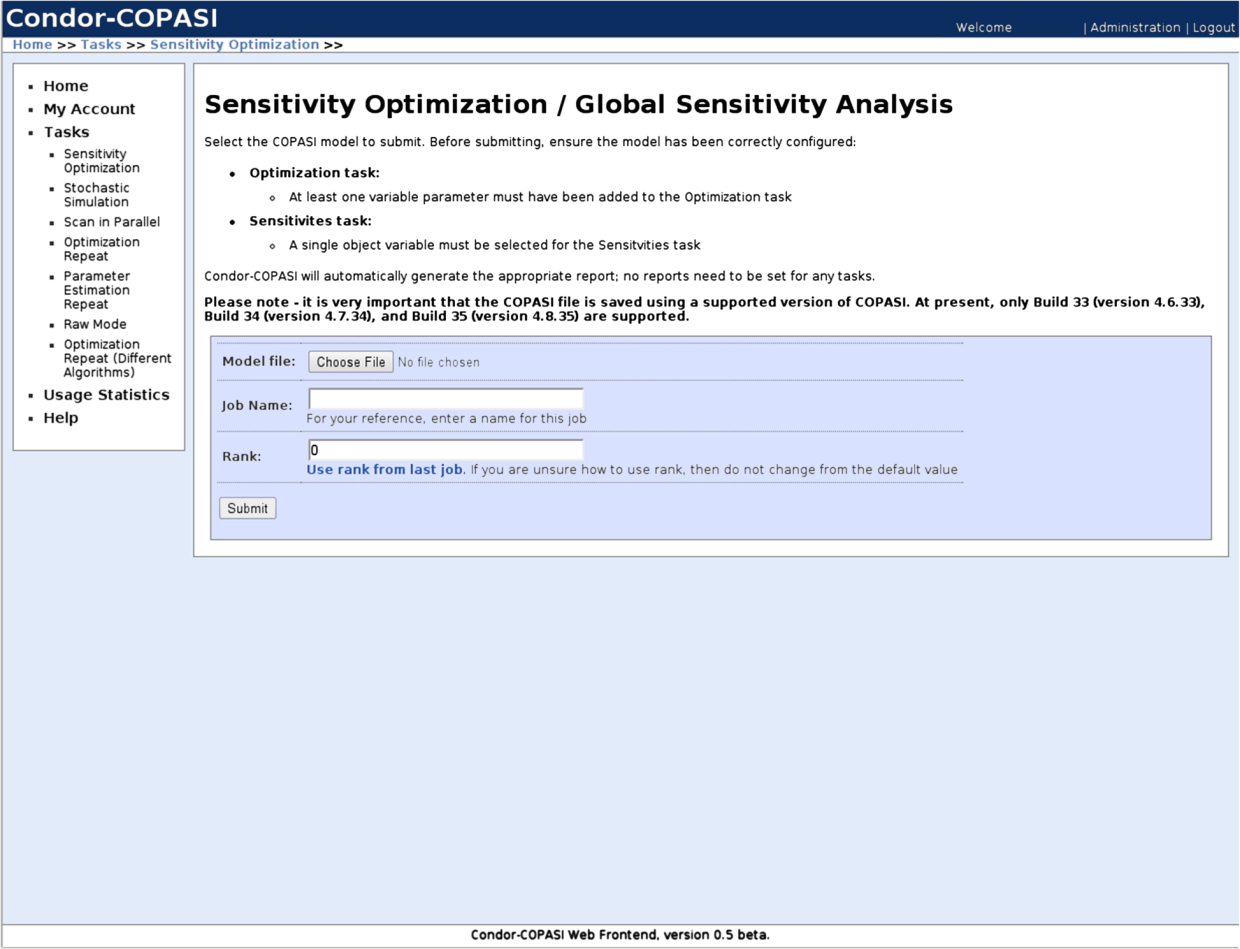
**Screenshot of job submission process. **A screenshot of the submission process for the global sensitivity analysis task. Other than preparing a COPASI model file, very little user interaction is required – all aspects of the Condor submission process are handled automatically.

Condor-COPASI allows users to submit a number of predefined tasks, each of which is amenable to running in parallel (see Use Cases for full details). Tasks are submitted by uploading a pre-prepared COPASI model using the web interface (Figure 1). Condor-COPASI then automatically determines the best way to split the task into parallel, automatically creates the necessary files, and submits the parallel jobs to Condor.

While the web interface is written in Python, all simulation and analysis tasks are performed using the COPASI Simulation Engine. Condor-COPASI works by automatically modifying the XML-based COPASI file format, generating a custom COPASI model file for each parallel job. In addition to the model file, a Condor job specification file is automatically generated for each parallel job. These files are submitted to the Condor pool, along with a copy of the COPASI Simulation Engine (CopasiSE) binary for the appropriate machine architecture. The COPASI Simulation Engine carries out all computation on the remote machine, writing output to a text file. These text files are transferred back to the Condor-COPASI server, where they are processed and collated.

**Figure 2:**
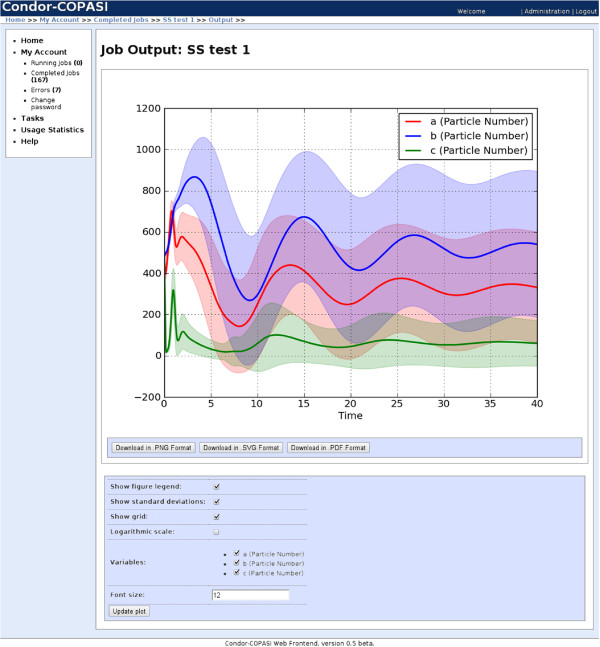
**Stochastic simulation output screenshot. **Screenshot showing showing the output of a stochastic simulation repeat task. For each of the three chemical species, particle number means and standard deviations are displayed.

**Figure 3:**
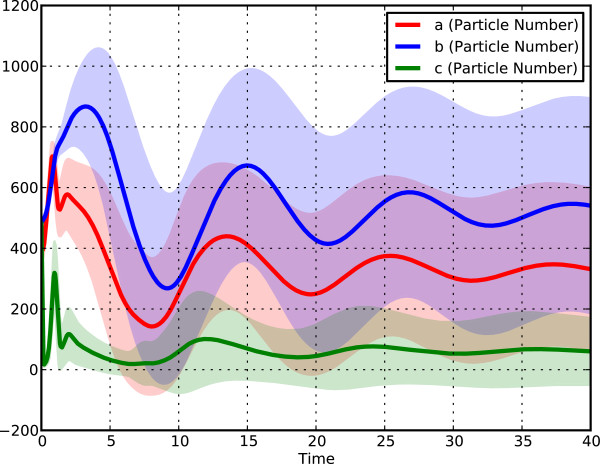
**Enlarged stochastic simulation output plot. **An enlarged version of the plot shown in Figure 2.

Various graphical plots can be produced. Static two-dimensional charts such as depicting the dynamics of mean and standard deviation of particle numbers in stochastic simulations are generated using the Python Matplotlib library [14] (Figure 2, Figure 3). An interactive bar chart, showing maximal and minimal sensitivity values of various model parameters for different potential parameter sets is also available. A scroll bar can be dragged to change the range of the parameter set, while the chart values update automatically. This feature is provided using the Google MotionChart API [15], the chart is displayed using a mixture of javascript and flash; all data is processed and rendered locally, and no data is sent to Google.

## Results

### Available use cases

Condor-COPASI currently provides access to seven use cases, each of which is likely to require significant amounts of computational power, and can be efficiently split into smaller independent parallel jobs.

#### Global sensitivity analysis

The global sensitivity analysis procedure, as described in [16], involves performing a number of optimizations – one maximization and one minimization for each parameter. Since each optimization can be run independently, the task can be trivially split, with one parallel job for each maximization or minimization. For example, a model with 30 parameters for which a sensitivity is to be calculated will generate 60 parallel jobs.

COPASI provides access to a number of optimization algorithms, including deterministic such as Truncated Newton [17] and stochastic such as Particle Swarm [18]. Any of these algorithms can be selected for use in this task. If a user wishes to try more than one optimization algorithm, multiple tasks should be prepared and submitted, each with a different algorithm selected.

Results are provided in table format, and through graphical summaries of the importance of each parameter to the target property of the model. Charts showing progress of the optimization tasks can also be generated, displaying the best optimization value against the number of steps taken by the algorithm.

#### Stochastic simulation repeat

This task allows for multiple repeats of a stochastic time-course simulation to be performed – a necessary procedure to determine the distribution of the trajectories. The results of each repeat are recorded, and for each time point particle number means and standard deviations are calculated. Plots of the results can be displayed in the web interface, and all result outputs can be downloaded as a tab-separated text file. Splitting of this task is carried out using the load balancing algorithm described below.

#### Parallel scan

The Parameter Scan task in COPASI automatically scans through various values for one or more parameters, performing a subtask such as steady-state analysis, a time-course simulation, or an optimization for each set of parameters. It can also be used to repeat a subtask a number of times using the same parameter values, or to sample parameter values from a random distribution. Condor-COPASI can split this task into parallel, using a non-overlapping range of parameter values for each chunk. The number of subtask repeats to perform in each parallel job is determined using the load balancing algorithm.

Due to the diverse nature of possible outputs from this task, Condor-COPASI does not produce graphical plots. Instead, a text-based output must be prepared for the task by the user, before the task is submitted. After the task has finished, a collated text file containing the output from all parallel jobs is made available to download; this file is identical to the output that would have been produced had the parameter scan run on a single machine.

#### Optimization repeat

Many of the optimization algorithms available in COPASI are stochastic, and will return a different solution each time they are run. Condor-COPASI can repeat an optimization multiple times, using the same algorithm for each repeat, and from these repeats, determine the best objective value and associated parameter set. The number of optimization repeats to be performed for each parallel job is determined using the load balancing algorithm. Once the task has finished running, a COPASI model file containing the best parameter values can be downloaded. Alternatively, the parameter values can be displayed in the browser.

#### Parameter estimation repeat

The Parameter Estimation task in COPASI is able to find the best parameter set to fit experimental data. Like the optimization task, many of the algorithms available are stochastic, and each time they are run will return a different solution. Condor-COPASI can repeat a parameter estimation multiple times, using the same algorithm for each repeat, determining the best solution from all repeats, using the load balancing algorithm to determine how many repeats to perform in each parallel job. Once the task has completed, a COPASI model file containing the best parameter set found by the parameter estimation can be downloaded, or the parameter values can be displayed on-screen.

#### Optimization with different Algorithms

Since COPASI has many optimization algorithms available, and it is not clear which one works best for each problem, a modeller often wishes to run the problem through several of them. Condor-COPASI can run an optimization several times using a different algorithm for each one. The user can select which algorithms will run, and can configure all tuning parameters of those algorithms. Each optimization is run as a separate parallel job, and after all jobs have completed, the best algorithm(s) are determined. In addition, a COPASI model file containing the best available parameter set as determined by the optimization can be downloaded, or the parameter values viewed in the browser.

#### Raw mode

The raw mode task is designed for advanced users, and allows one or more COPASI tasks to be repeated an arbitrary number of times. The user is able to specify all command-line arguments for the CopasiSE binary, and must specify any required input and output files. One repeat is performed per parallel job, and any output files generated must be manually collated and processed by the user. This mode extends the applications of Condor-COPASI to a number of other possibilities, however it requires the user to understand the use of COPASI through the command line interface, as well as a basic knowledge of distributed computing.

### Load balancing

The Stochastic Simulation, Parallel Scan, Optimization Repeat and Parameter Estimation Repeat use cases all involve repeating a particular task multiple times, and can be run in parallel by performing a certain number of repeats per job. For these tasks, the user specifies the total number of repeats to perform, while the number of repeats to perform per job, and subsequently the total number of parallel jobs, is determined using a load balancing algorithm.

The load balancing algorithm constructs the parallel jobs such that they each run for an approximately equal length of time, *t*, a parameter which is set by the system administrator. The algorithm first measures how long a single repeat of the task to be performed takes to complete. The parallel jobs are then built up with an appropriate number of repeats, such that the total run time of each job is approximately equal to *t*. If a single run takes longer than *t*, the algorithm will time out, and assign only one repeat per job.

It is important to choose a good value for *t*. If it is too small, each task will produce a large number of parallel jobs, each with few repeats. In this situation, the overhead associated with submitting jobs to the Condor pool will become a significant factor, and in an extreme situation, running the job on Condor could take longer than if all jobs had been run sequentially on a single machine. However, if *t* is too large, then each task will produce a small number of parallel jobs, each taking a long time to complete. In this situation, the computational capacity of the Condor pool will not be fully exploited, and the benefits of running in parallel will be negated. In addition, jobs may run for too long, risking eviction from the machines they are running on – non-dedicated machines in the pool are normally only available when they would otherwise be idle, such as overnight and at weekends – meaning there is often an upper bound on the length of time a job can run for.

**Figure 4:**
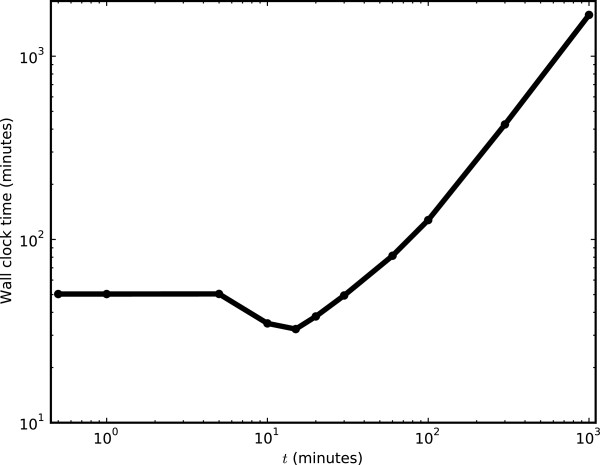
**Tuning the load balancing algorithm. **The load balancing algorithm uses a parameter *t *to determine, where possible, the ideal run time for a single parallel job. We determined that the optimal *t *value, minimizing the wall clock time for the task, was 15 minutes. At *t * < = 5 minutes, only one repeat was performed per parallel job, and the job submission overhead outweighed the benefits of running in parallel. At *t * > = 20 minutes, too many repeats were performed by each parallel job, and the benefits of running in parallel were reduced. Our tests were performed by running 1500 repeats of an optimization task, where each optimization had a 5 minute run time, on a Condor pool containing 2000 available executing nodes.

To determine the best value for *t*, we ran an optimization repeat task with *t* values ranging from 0.1 to 1000 minutes (Figure 4). We found that setting *t* to 15 minutes gave a good trade-off between job submission overhead and gaining the benefits of running jobs in parallel. However, factors such as the number of machines in the Condor pool, and the speed of network communications between the server and machines in the pool will impact on this value. Therefore, we advise administrators to consider adjusting this value if necessary.

We also include an option for applicable tasks to override the load balancing algorithm, and to construct jobs with only one repeat per parallel job. This is useful in situations where the user knows *a priori* that each repeat is likely to take longer than *t*, saving them from having to wait for the load balancing algorithm to time out, or in situations where one wishes to make the run-time for each parallel job as short as possible.

### Error handling

Condor handles various types of error – if jobs are evicted from the machine they are running on, they will automatically be re-queued and executed on an available machine. In cases where a job fails (for example, due to a malformed job specification file), the job will remain in the queue, but will be marked as ‘held’. We note that while Condor supports application checkpointing for compatible software, which allows evicted jobs to resume on a different machine without loss of computation time, COPASI does not support checkpointing, so all evicted jobs must begin again from the start.

Condor-COPASI monitors the status of each submitted job – the queue of jobs is periodically polled to check for jobs marked as ‘held’, and the exit status of all completed jobs is checked (a non-zero exit status indicates an error) by parsing the log files. When an error is detected, Condor-COPASI will try to determine whether it happened before COPASI was executed on the remote machine (indicating a malformed job specification file) or after (indicating a problem with the way the task was set up in COPASI).

**Figure 5:**
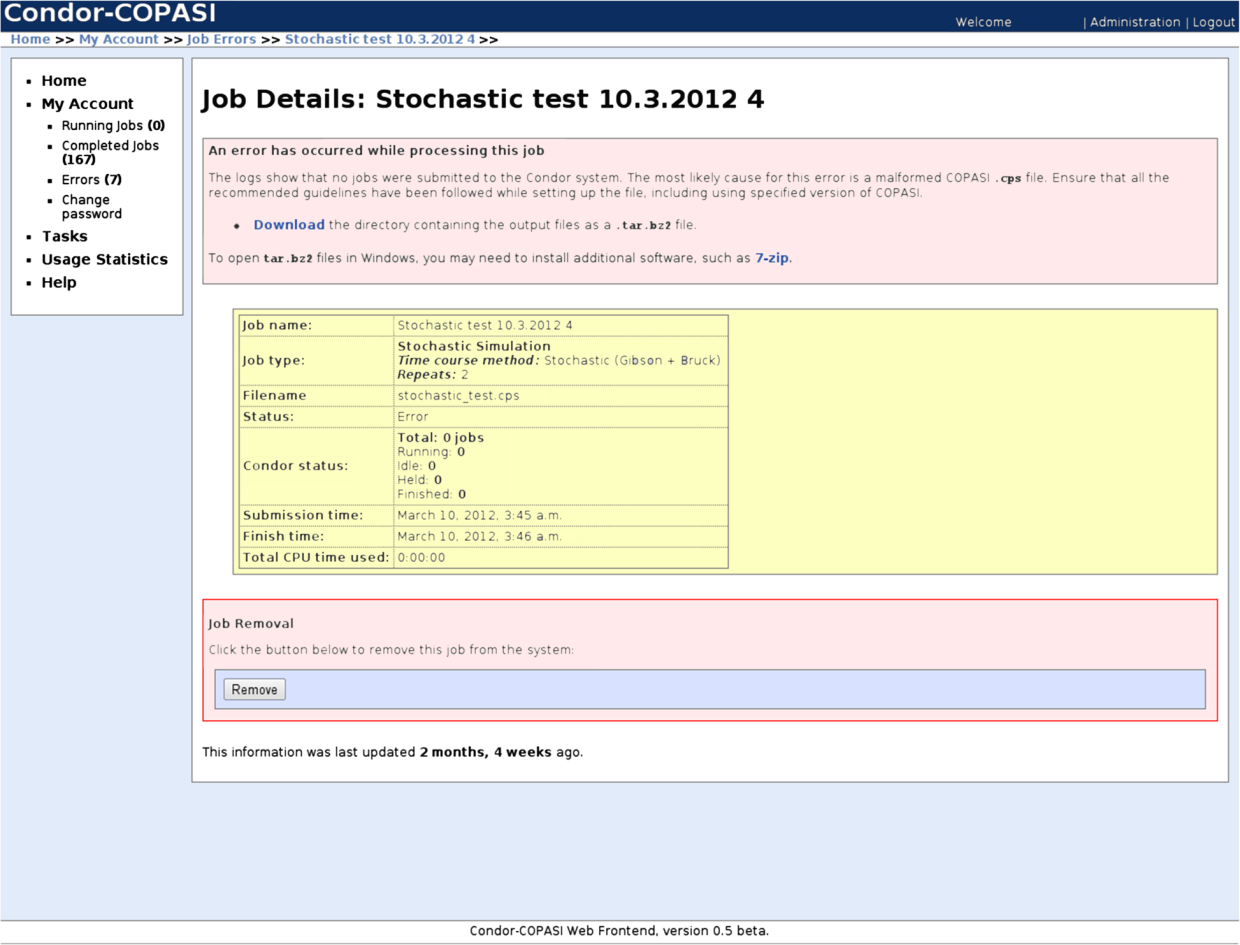
**Screenshot of failed job submission. **A screenshot of a task which failed to run on Condor. Condor-COPASI is able to handle failed jobs, and can analyze log files to determine the probable cause of failure, along with suggestions on how to fix any problems.

After detecting an error, Condor-COPASI will email the user to notify them that the task did not complete successfully. The web interface will display the probable cause of the job failure (Figure 5), and allow the user to download a compressed copy of all files generated by Condor-COPASI, including Condor log files, automatically generated COPASI model files, and any returned results files. Such files can assist the user in determining the cause of the failure, and allow them to manually collate any results generated if they will be useful.

Finally, Condor-COPASI will log all activity to a text file, including any errors and exceptions encountered. Examining this file can be helpful to determine the cause of a failure if it is otherwise not clear.

### Performance

To illustrate the effectiveness of Condor-COPASI, we collated data from 12 months of real-world usage on our installation of Condor-COPASI; for each task submitted, we recorded the total CPU time used by all parallel jobs, the wall clock time of the task, and the number of parallel jobs used. The cumulative total of CPU time used does not include any time spent queuing for resources to become available, or any other delays caused by the Condor job submission process. It is analogous to the time it would take to run each parallel job sequentially on a single-core local machine, and therefore provides a good measure of how long the computing task would have taken to perform without using Condor-COPASI. The wall clock time represents the total waiting time between submission of the task to Condor-COPASI and its completion, and includes time where possibly no jobs were running because the Condor pool was running jobs for other users. Therefore, the total waiting time for each task is not necessarily dependent only on the nature and size of the task. The wall clock times reported come from a production system where Condor was shared with other users and are therefore indicative of typical usage.

In all cases, the tasks were run on our local Condor pool of approximately 2000 execute nodes. The most common hardware configuration in the pool is an Intel Core2 Quad processor at 3GHz, with 4GB of RAM. However, we note that the pool is heterogeneous, with a number of different hardware configurations, and nodes continuously coming online and offline.

**Figure 6:**
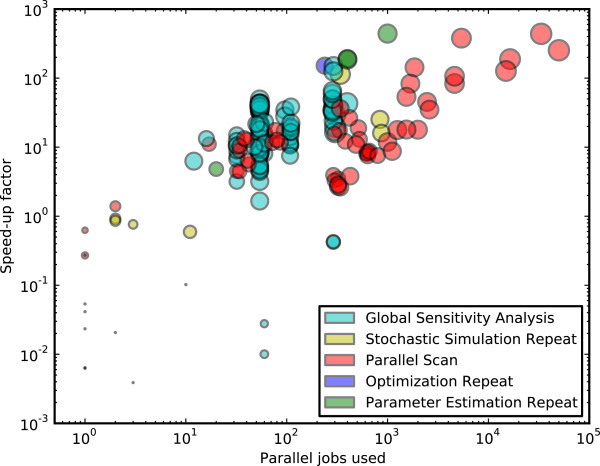
**Speed-up Factors. **The speed-up factor (CPU time/wall clock time) and number of parallel jobs used were recorded for 190 tasks submitted during 12 months of real-world usage on our Condor-COPASI installation, with a Condor pool of approximately 2000 execute nodes. The color of each marker indicates the type of task performed, and the area of each marker is proportional to the base-10 logarithm of the CPU time used.

To illustrate the improvements in run time using Condor-COPASI, we calculated the speed-up factor for each task, defined as CPU time/wall clock time. The speed-up factors were plotted against the number of parallel jobs used (Figure 6). In general, speed-up factors of between 10^0^ and 10^3^are seen. For tasks except global sensitivity analysis, there is a roughly linear relationship between the number of parallel jobs used, and the speed-up factor achieved. We note that the global sensitivity analysis task cannot be parallelized as efficiently as the other task types, so the highest speed-up factors for this task type were lower than other task types. Some tasks achieved a low speed-up factor ( < 10^0^); most of these tasks had a low CPU time and used few parallel jobs, so there was little benefit to running in parallel. Of particular note are two global sensitivity analysis tasks with speed-up factors of around 0.1 and 0.3, with 60 parallel jobs each. For these tasks, the CPU time used was very low (around 6 minutes), while several hours were spent waiting for resources in the Condor pool.

### Specific examples

To further illustrate the benefits of running tasks on Condor-COPASI, we describe 5 detailed examples of tasks that were run (summarized in Table 1), showing the extent to which they were parallelized, and the speed-up factor achieved (defined as CPU time/wall clock time).

**Table 1:** Example tasks run on Condor-COPASI on a pool of 2000 nodes, showing wall clock time on Condor, total CPU time, and speed-up factor (CPU time/wall clock time)

**Task type**	**Description**	**Number of parallel jobs**	**Wall clock time**	**CPU time used**	**Speed-up factor**
Global sensitivity analysis	27 parameters from NF *κ *B signalling model [19]	54	7 hours	368 hours	53
Global sensitivity analysis	30 parameters from MAPK signalling model [20]	60	7 hours	90 hours	13
Stochastic simulation	1,000,000 repeats of calcium oscillation model [21]	340	20 hours	2,280 hours	114
Parallel scan	300,000 repeats of NF *κ *B signalling model [19]	14,926	31 hours	3,980 hours	128
Parallel scan	100,000 repeats of MAPK signalling model [20]	1,849	3 hours	429 hours	143

Global sensitivity analysis – we ran a global sensitivity analysis on a model of NF*κ* B signal transduction [19], examining the control of 27 parameters on the frequency of nuclear NF*κ* B oscillation, using a parameter space consisting of the original parameter values ±20*%*. The task completed on Condor-COPASI in approximately 7 hours using 54 parallel jobs, using a cumulative total of 368 hours of computing time, achieving a speed-up factor of 53.

Global sensitivity analysis – we ran a global sensitivity analysis on a model of the MAPK signalling cascade [20], examining the control of 30 parameters on the concentration of nuclear MAPK-PP, using a parameter space consisting of the original parameter values ±50*%*. The task completed on Condor-COPASI in approximately 7 hours using 60 parallel jobs, using a cumulative total of 90 hours of computing time, achieving a speed-up factor of 13.

Stochastic simulation repeat – we ran 1,000,000 repeats of a stochastic time-course simulation of a three-variable calcium oscillation model [21]. Condor-COPASI completed the task in approximately 20 hours (including time taken queuing for resources and processing the resulting output files), using a cumulative total of 2,280 hours of computing time across 340 parallel jobs, achieving a speed-up factor of 114.

Scan in parallel – we used the parallel parameter scan task to perform 300,000 Monte Carlo simulations of an NF*κ* B signal transduction model [19]. Condor-COPASI completed the task in approximately 31 hours, using 14,926 parallel jobs, using a cumulative total of 3,980 hours of computing time, achieving a speed-up factor of 128.

Scan in parallel – we used the parallel parameter scan task to perform 1,000,000 Monte Carlo simulations of a MAPK signalling cascade model [20]. Condor-COPASI completed the task in approximately 3 hours, using 1,849 parallel jobs, using a cumulative total of 429 hours of computing time, achieving a speed-up factor of 143.

## Discussion

### Performance

Condor-COPASI enabled us to significantly reduce the run time of many simulation and analysis tasks. In 12 months of real-world usage on our installation, we saw tasks running up to 442 times faster than if they had been run on a single computing core, with an average speed-up of 32 times. This has enabled us to perform model simulations and analyses that would otherwise not have been feasible, with some individual analysis tasks using more than a year of computing time, but completing in less than a day.

In an ideal situation, for most task types, the decrease in run time for running a particular task on Condor compared to running it on a single computing core should be proportional to the number of executing nodes available in the Condor pool. So, for example, if we have a task that takes 1000 minutes to run on a single core, and a Condor pool available with 1000 equally fast executing nodes, then the speed increase would be 1000-fold and the task would complete in 1 minute.

In practice, various limitations, overheads and discrepancies in the local network architecture and Condor pool mean the overall running time for a task will always be more then the theoretical minimum, and it is rarely possible to predict exactly how long a task will take to complete.

There is an overhead associated with submitting and running each parallel job – the Condor Master must add the job to the queue for resources and assign it to an appropriate executing node when one becomes available. The submitting node must then send the associated model and data files, along with a copy of the COPASI binary, over the network before job execution can start. In a situation where we have a small number of jobs to submit, each of which will take a long time to execute, the overhead will likely not be significant. However, if we have a large number of jobs to submit (especially in the situation where there are more jobs than executing nodes available to run them), each of which will can be executed in a short amount of time, then the submission overhead will be more significant, and could become a limiting factor in the execution time for the task. In this situation, it may be preferable to construct the parallel jobs so that they each perform a certain number of repeats, increasing the job execution time while keeping the submission overhead constant, thus reducing the impact of the submission overhead. The load balancing algorithm described above attempts to find an ideal compromise between the degree to which the job is parallelized and the job submission overhead.

Another factor that can affect the execution time of our jobs is the potentially heterogeneous nature of the machines in the Condor pool – disparities in hardware specification will mean that the job execution time will vary from machine to machine. Thus, unless we specify exact hardware requirements for our jobs, their execution time will vary depending on the specification of the machine they are assigned to.

We must also consider that we may be competing for the available resources with other users. Therefore, the number of available executing nodes will depend on how many other users are using the Condor pool, and our priority within the queue relative to others.

Finally, the extent to which we can parallelize our task depends on the task type. Where the task involves repeating a subtask a certain number of times, we can parallelize up to the extent where we have one repeat per parallel job. Other task types, such as the global sensitivity analysis, can only be parallelized according to the number of parameters we are investigating. For example, an analysis on a model with 10 parameters will produce 20 parallel jobs (one maximizing optimization and one minimizing optimization for each parameter). In this situation, having more than 20 executing nodes available will not speed up overall job execution any more than having just 20.

In summary, the degree to which running a task on Condor will speed up execution compared to running the same task on a single local machine depends on the type of task being performed, and the degree to which it can be parallelized, and will also depend on a number of other factors, such as submission overheads and demand for the available resources. However, in testing, we saw vast improvements in the run-time of all task types (see Table 1).

### Comparison with existing software

Unlike tools such as COPASI Web Services, Condor-COPASI works as a standalone piece of software, performing all aspects of file preparation, simulation and results processing with a user-friendly interface, requiring minimal user interaction. Other tools, such as VCell and JWS Online, provide user-friendly graphical interfaces, but are less able to fully exploit the parallel nature of a distributed computing pool.

### Other cluster management tools

Condor is one of a number of systems designed for cluster management and job scheduling; other systems with widespread deployment include Oracle Grid Engine (formally known as Sun Grid Engine) [22], Maui [23], and PBS [24,25]. The strengths of Condor (namely cross-platform support including Windows, Linux and OSX, its ability to utilize non-dedicated resources, and its fully open-source nature), make it an attractive choice for academic institutions, particularly those looking to utilize existing hardware. However, we recognize that many potential users may only have access to other distributed computing systems. If demand dictated, it could be possible to add support for other systems at a later date, though differences in job preparation, submission, and monitoring would make this task non-trivial. As an alternative, we note that it is possible to install Condor alongside other job schedulers; the Condor scheduler will only assign jobs to computing nodes with available computational capacity.

We also note the growing popularity of cloud computing systems, such as Amazon’s Elastic Compute Cloud (EC2), which allow users to lease any required computational capacity by the hour, without having to invest in dedicated hardware. Such systems are particularly appealing to researchers who need only occasional access to large amounts of computational power. It is currently possible to use Condor on Amazon EC2 by running one or more EC2 instances as executing nodes. However, setting up such an arrangement is difficult, requiring a detailed knowledge of network configuration, and is likely to be beyond the capacity of most casual users. Therefore, a possible future extension of Condor-COPASI could be to automatically configure and launch EC2 or other cloud computing instances to form a Condor pool, and to use this to complement, or as an alternative to, a local Condor pool.

## Conclusions

Condor-COPASI enables the use of ubiquitous distributed computing by making it easy to submit systems biology simulation and analysis tasks without requiring any knowledge of programming or managing networks of workstations. The computational power available in such pools of computers can be vast, particularly in institutions such as Universities, which have thousands of computers serving as terminals for only a portion of the day. Being able to efficiently utilize such a resource can enable large-scale simulations and analyses to be performed that would otherwise require too much computing power to be feasible.

## Availability and requirements

*Project name:* Condor-COPASI

*Project home page:*http://code.google.com/p/condor-copasi/

*Operating system:* Server-side software: Linux; user interface can be accessed on any operating system through a modern web browser

*Programming language:* Python and Django

*Other requirements:* Python 2.6; Django 1.2; a web server with Python support such as Apache; copies of the COPASI Simulation Engine (CopasiSE) binary in 32-bit and 64-bit mode for Windows, OSX and Linux; the LXML and Matplotlib Python libraries; Condor version 7.4 or higher, a database application – either MySQL, PostgreSQL, SQLite or Oracle – with an appropriate Python wrapper.

*License:* Artistic License 2.0

*Restrictions to use by non-academics:* None

Full instructions on deployment, and an instruction manuals users are available on the project home page: http://code.google.com/p/condor-copasi.

## Competing interests

The authors declare that they have no competing interests.

## Authors’ contributions

EK wrote the software and prepared the first draft of the manuscript. PM and EK developed the specifications of the software. PM, EK and SH tested the software. All authors read, improved, and approved the final manuscript.

## Funding

Pfizer/BBSRC grant BB/G529859/1, NIH grant R01 GM090219, BBSRC/EPSRC grant BB/C008219/1 (MCISB), EU FP7 grant 201142 (UNICELLSYS).
